# Experience with luspatercept therapy in patients with transfusion-dependent low-risk myelodysplastic syndromes in real-world clinical practice: exploring the positive effect of combination with erythropoietin alfa

**DOI:** 10.3389/fonc.2024.1398331

**Published:** 2024-10-02

**Authors:** Anna Jonasova, Slavka Sotakova, Petra Belohlavkova, Lubomir Minarik, Tomas Stopka, Jan Jakub Jonas, Tatiana Aghova, Zuzana Zemanova

**Affiliations:** ^1^ First Department of Medicine, Hematology, General University Hospital and First Faculty of Medicine, Charles University, Prague, Czechia; ^2^ 4th Department of Internal Medicine—Hematology, Charles University Hospital, Hradec Kralove, Czechia; ^3^ BIOCEV, First Faculty of Medicine, Charles University, Prague, Czechia; ^4^ University College of London, London, United Kingdom; ^5^ Center of Oncocytogenomics, Institute of Clinical Biochemistry and Laboratory Diagnostics, General University Hospital and First Faculty of Medicine, Charles University, Prague, Czechia

**Keywords:** myelodysplastic syndromes, anemia, luspatercept, epoetin alfa, treatment

## Abstract

**Background:**

Luspatercept, an inhibitor of the transforming growth factor beta (TGF-β) pathway, is a novel treatment for anemic patients with lower-risk myelodysplastic syndromes (MDS) with transfusion dependence (TD) who do not respond to erythropoiesis-stimulating agents (ESA) therapy or are not suitable candidates for this treatment. We present real-world experience with luspatercept therapy from two hematology centers in the Czech Republic.

**Methods:**

By January 2024, 54 MDS patients (33 men, 21 women) with a median age of 74 years (range, 55–95) were treated with luspatercept ± ESA at two Charles University hematology centers in Prague and Hradec Králové. According to the WHO 2016 classification, the cohort included 32 MDS-RS-MLD, seven MDS-MLD, two patients with 5q- + ring sideroblasts (RS), 12 RARS-T, and 1 patient with CMML-0 + RS. *SF3B1* mutation data were available for 45 patients. All patients were in the IPSS-R and IPSS-M lower-risk groups (except four IPSS-M high). The median follow-up was 17 months (range, 1–54). All patients were transfusion-dependent. Thirty-five (64.8%) patients had a high transfusion burden (HTB) with ≥ 4 transfusion units (TU)/8 weeks, and 19 (35.2%) had a low transfusion burden (LTB) (< 4 TU/8 weeks). The median time between diagnosis and initiation of luspatercept was 27 months (range, 4–156). ESA were used prior to luspatercept in 45 patients, and luspatercept was used as first-line treatment in nine patients. Thirty-one (61%) patients were treated simultaneously with ESA.

**Results:**

Only patients who received luspatercept for ≥ 8 weeks (51 patients) were assessed. We evaluated the achievement of transfusion independence (TI) lasting 8, 12, 16, and 24 weeks. Thirty-two (62.7%) patients achieved TI for ≥ 8 weeks, 31 (60.7%) for ≥ 12 weeks, 29 (56.8%) for ≥ 16 weeks, and 25 (49%) for ≥ 24 weeks. Hematologic improvement (HI) without TI was achieved in six patients (11.7%). Overall, HI + TI was achieved in 38 patients (74.5%). Epoetin alfa was used simultaneously in 31 patients (60.7%). In 21 (55.2%) of all responding patients, concomitant therapy with epoetin alfa led to an improved response, with 16 reaching TI. Thirteen (25.5%) patients were nonresponders. Eight (21%) patients experienced therapy failure and became transfusion-dependent again. Optimal response required a gradual increase in the luspatercept dose to 1.75 mg/kg in up to 35 patients, with 23 responders (TI + HI). Response rates varied by transfusion burden: 79% in LTB and 50% in HTB reached TI. Of RS+ patients, 70% reached TI, while only one out of five RS− patients achieved TI. Among 39 *SF3B1*-positive patients, 61.6% achieved TI. In the low and very low IPSS-M groups, 86% of patients responded (TI + HI), compared to 62% in the moderate-low group. Luspatercept was well-tolerated, with no adverse events higher than grade II toxicity.

**Conclusion:**

We have demonstrated in real-world clinical practice that luspatercept is a very effective agent, even in an unselected, pretreated, significantly TD MDS population. The effect was particularly high in the IPSS-M low and very low groups. We believe that the relatively high response rate in our patients was influenced by the frequent use of a higher dose (1.75 mg/kg) and especially by adding ESA to luspatercept in poorly responding patients.

## Introduction

Myelodysplastic syndrome (MDS) is a heterogeneous group of myeloid malignancies characterized by the clonal proliferation of malignant stem and progenitor cells, cytopenia in peripheral blood, and an increased risk of progression to acute myeloid leukemia. In 2022, the World Health Organization (WHO) reclassified MDS as myelodysplastic neoplasia (MDN). New subgroups are also defined based on genetic or molecular genetic abnormalities.

Regarding therapy choice, MDS patients are divided into lower and higher-risk groups, aided by the International Prognostic Scoring System-Revised (IPSS-R), which combines multiple factors (1). The advent of molecular genetics, with next-generation sequencing and the identification of a set of mutations of more or less prognostic significance, has led to the development of the new IPSS-molecular (IPSS-M) scoring system (2).

Approximately 70% of patients diagnosed with MDS are classified as lower risk. The goals of therapy for low-risk patients are to eliminate or reduce cytopenia, improve quality of life, and prolong overall survival (OS). In treating low-risk patients, anemia is the predominant problem. Profound anemia significantly impacts the quality of life. Overall, around 90% of MDS patients are anemic, and 60% require transfusions sooner or later. Meanwhile, transfusion dependence and its severity are another factor that not only reduces the quality of life but also increases morbidity and mortality in MDS patients (3). Transfusion dependence is also associated with iron overload, leading to subsequent organ damage (4). Therefore, current goals of treatment for lower-risk MDS include achieving independence from transfusions, increasing hemoglobin levels, and restoring effective erythropoiesis.

The standard first-line treatment for symptomatic anemia in patients with lower-risk MDS non-5q- is erythropoiesis-stimulating agents (ESA) (5, 6). Unfortunately, many patients treated with ESA do not respond, and most patients who do respond eventually relapse and become dependent on transfusions. Until recently, treatment options were limited for patients who were not eligible for ESA or for whom ESA has failed. A recent breakthrough in treating anemia in low-risk MDS patients is the use of transforming growth factor beta (TGF-β) pathway blockers.

TGF-β pathway blockers include luspatercept and galunisertib. Luspatercept is a modified IIB receptor activin that inhibits the SMAD2/3 pathway by blocking growth differentiation factor 11 (GDF11—ligand of the TGF beta pathway) and leads to the potentiation of erythroid lineage differentiation and proliferation in the late stages of erythropoiesis (7). Luspatercept was approved in 2022 by the European Medicines Agency for treating anemic low-risk MDS patients with ring sideroblasts (MDS-RS-MLD, MDS-RS-SLD, and RARS-T according to the 2016 WHO classification) with transfusion dependence who are unresponsive or unsuitable for erythropoietin therapy. Three clinical trials have made important contributions. A phase II study (PACE-MDS) was a multicenter, open-label, dose-finding study that enrolled 58 patients (8). Transfusion independence (TI) was achieved in 38% of patients. Hematologic improvement (HI) according to the 2006 IWG criteria (9) was achieved in 63% of patients. The best responders were patients with ring sideroblasts (RS) or SF3B1 mutation positivity, in whom HI was achieved up to 77%.

Due to the significant efficacy and good tolerability of luspatercept, the drug was subsequently investigated in patients with MDS RS+ in the multicenter, randomized, double-blind, placebo-controlled phase III MEDALIST trial (10). In luspatercept-treated patients, TI was achieved in 38%. Patients with ring sideroblasts and thrombocythemia, a distinct subunit according to the WHO (RARS-T), responded significantly well (80% response rate). Importantly, luspatercept may also have trilineage activity (11). The most recent clinical trial, COMMANDS, proved luspatercept’s superiority to ESA, with almost 60.4% TI (Garcia-Manero et al. ASH 2023, abstract No. 193) lasting more than 12 weeks in luspatercept-treated low-risk anemic patients versus only 31% TI in erythropoietin-treated patients (12). Here, we present the real-world experience of two hematology university centers in the Czech Republic.

## Patients and methods

By January 2024, 54 low-risk MDS patients (33 men, 21 women) with a median age of 74 years (range, 55–95) were treated with luspatercept with or without epoetin alfa at two locations: the I. Medical and Hematology Department at Charles University General Hospital in Prague (45 patients) and the 4th Department of Internal Medicine - Hematology at Charles University Hospital in Hradec Králové (nine patients). Patient characteristics are summarized in **Table 1**. According to the WHO 2016 classification, there were 32 patients with MDS-RS-MLD, seven with MDS-MLD, two with 5q- + ring sideroblasts (RS), 12 with RARS-T, and one with CMML-0 + RS. SF3B1 mutation information was available for 45 patients.

**Table 1:** Characteristics of patients.

Characteristics	Numbers
Number of treated	54
Men/women	33/21
Age median (range)	74 (55–95)
Hb level median (range)	81 g/L (55–84)
Epo level median (range)	374 IU/L (150–770)
Ferritin level median (range)	1,307 μg/L (185–3,890)
Dg WHO 2016	
MDS-RS-MLD	32
MDS-MLD	7
RARS-T	12
5q- + RS	2
CMML + RS	1
WHO 2022 (*SF3B1* known in 45 pts)	
MDS-*SF3B1*	27
MDS with 5q- (+RS and *SF3B1*)	2
MDS-LB	11
Hypoplastic MDS	1
MDS/MPN	
CMML (+ RS)	1
MDS/MPN *+ SF3B1*	11
MDS/MPN not otherwise specified (+RS and thrombocythemia)	1
IPSS-R	
Very low	6
Low	42
Intermediate	6
High and very high	0
IPSS-M (35 pts evaluated by NGS)	
Very low + low	15
Moderate low	9
Moderate high	7
High	4
Very high	0
Cytogenetic categories (IPSS-R)	
Very good	4
Good	38
Intermediate	11
Poor	1
Very poor	0
***SF3B1* mutation tested by NGS/PCR**	45
Positive	39
(Isolated *SF3B1* mutation—from NGS tested)	15
Negative	6
Missing	9

Hb, hemoglobin; Epo, erythropoietin; MDS-RS-MLD, myelodysplastic syndrome with multilineage dysplasia and ring sideroblasts; MDS-MLD, myelodysplastic syndrome with multilineage dysplasia; RARS-T, refractory anemia with ring sideroblasts and thrombocythemia; CMML, chronic myelomonocytic leukemia; MDS-LB, myelodysplastic syndrome with low blasts; MDS/MPN, myelodysplastic/myeloproliferative neoplasm; IPSS-R, International Prognostic Scoring System-Revised; IPSS-M, International Prognostic Scoring System-Molecular; NGS, next-generation sequencing; PCR, polymerase chain reaction.

Using the WHO 2022 classification, the breakdown was as follows: 27 patients with SF3B1, two with 5q- (+ SF3B1 and RS), 11 with MDS-LB (low blasts), one with hypoplastic MDS, one with CMML + RS, and 11 with MDS/MPN with SF3B1. There was one patient with MDS/MPN not otherwise specified. None of the patients had increased bone marrow blasts. According to the International Prognostic Scoring System Revised (IPSS-R), 54 patients were evaluated (including those with RARS-T). The IPSS-R categories were as follows: six very low, 42 low, and six intermediate risk (we did not treat high- or very high-risk patients). For the IPSS-M system, 35 patients with known next-generation sequencing (NGS) data were evaluated: 15 were very low + low, nine were moderately low, seven were moderately high, four high, and 0 very high.

Luspatercept was administered subcutaneously once every 3 weeks, starting at a dose of 1.0 mg/kg, which was increased stepwise to 1.33 mg/kg and then to a maximum of 1.75 mg/kg. Epoetin alfa was administered subcutaneously at doses of 40,000–80,000 IU weekly. The median follow-up from the beginning of luspatercept treatment was 17 months (range, 1–54). We included two patients who were originally in the COMMANDS study but were subsequently treated further outside the study. Our relatively short median follow-up time is influenced by the recent approval of luspatercept. The median duration of luspatercept treatment was 11 months (range, 1–54). The median time between diagnosis and the initiation of luspatercept therapy was 27 months (range, 4–156). All patients were transfusion-dependent. Transfusion dependency (TD) before luspatercept initiation ranged from 2 transfusion units (TU)/8 weeks to 12 TU/8 weeks. A total of 35 patients (64.8%) belonged to the high transfusion burden (HTB) group (≥ 4 TU/8 weeks), while 19 patients (35.2%) belonged to the low transfusion burden (LTB) group (< 4 TU/8 weeks) (**Table 2**). All patients were transfusion-dependent or refractory to erythropoietin-stimulating agents (ESAs). ESA was used before luspatercept in 45 patients, and luspatercept was used as a first-line treatment in only nine patients. All previous therapies are listed in **Table 2**. Eleven patients had undergone three lines of treatment.

**Table 2:** Treatment prior to luspatercept.

Treatment modalities	Number of patients (%)
**Transfusion dependency**	54 (100%)
Low (LTB) (< 4 TU/8 weeks)	19 (35.2%)
High (HTB) (≥ 4 TU/8 weeks)	35 (64.8%)
Previous treatment	
ESA (including ESA + other treatment)	45
ESA + G-CSF	5
ESA + prednisone	5
Study drug (for ESA refractory)	4
Lenalidomide ± ESA	4
AZA ± ESA	2
Cyclosporine + steroids ± ESA	1
Transfusion only (very high level of Epo)	5

LTB, low transfusion burden; HTB, high transfusion burden; ESA, erythropoiesis-stimulating agents; G-CSF, granulocytic colony-stimulating factor; AZA, azacytidine; Epo, erythropoietin.

## Results

**Figure 1:**
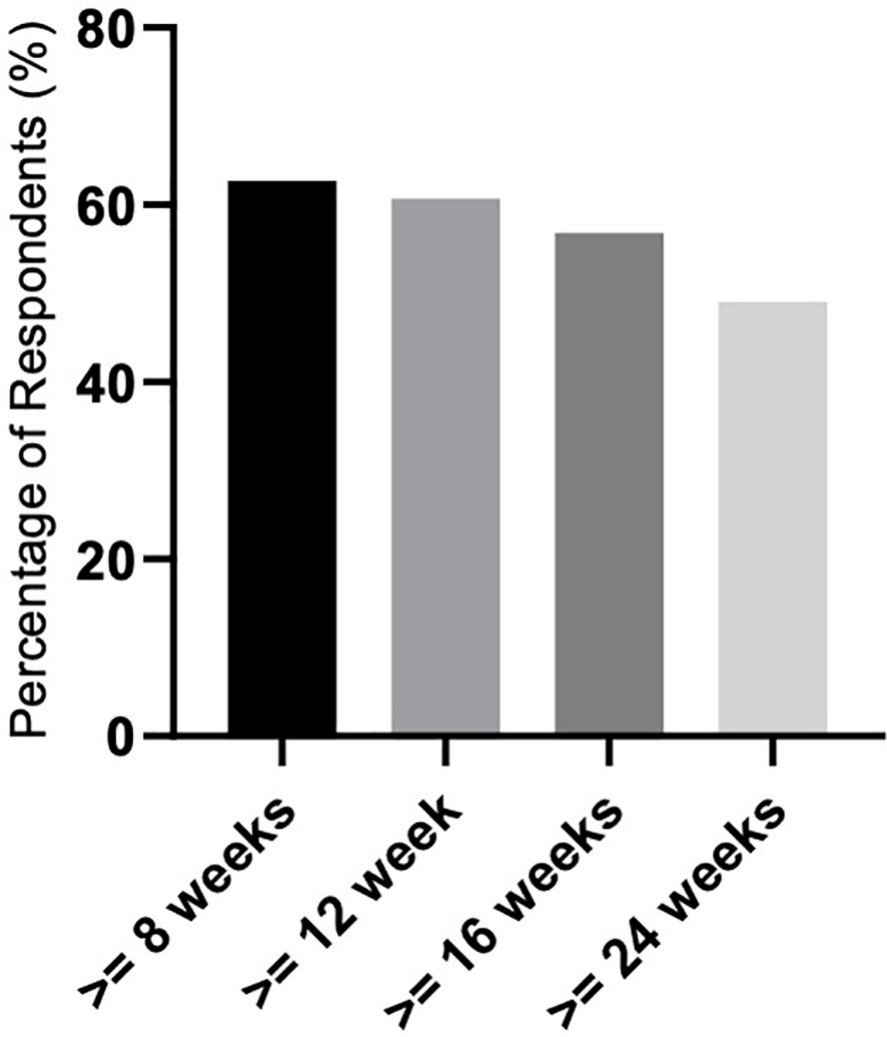
Response rate related to achieving transfusion independence (TI) lasting ≥ 8, 12, 16, and 24 weeks.

We evaluated 51 patients in terms of response. Only those who received luspatercept for more than 8 weeks were included in the final response evaluation to assess the achievement of transfusion independence (TI) or hematologic improvement (HI) according to the IWG criteria 2006. We evaluated the achievement of TI lasting 8, 12, 16, and 24 weeks during luspatercept therapy. Thirty-two (62.7%) patients achieved TI lasting ≥ 8 weeks, 31 (60.7%) for ≥ 12 weeks, 29 (56.8%) for ≥ 16 weeks, and finally, 25 (49%) patients achieved TI lasting ≥ 24 weeks (**Figure 1**).

Only hematological improvement without TI (according to IWG criteria 2006) was achieved in six patients (11.7%). Overall, the response (HI + TI) was achieved in 38 patients (74.5%).

Regarding the duration of response, the recent approval of luspatercept by the drug agencies should be taken into account. The duration of the response is affected by the shorter follow-up of patients still on luspatercept, except for one patient who followed for 54 months and was originally included in the COMMANDS series. Most of our patients are still on therapy, and many started luspatercept recently, so we do not report the median duration of response in our analysis. There were 13 (25.5%) nonresponding patients.

Epoetin alfa was used simultaneously in 31 (60.7%) patients. In eight patients, we used a dose of epoetin alfa of 40,000 units/week; in other patients, the dose was 80,000 units/week. In 21 (55.2%) of all responding patients, concomitant therapy with epoetin alfa led to an improved response, with 16 of them achieving TI and five HI (**Table 3**). To achieve an optimal response, we had to gradually increase the dose of luspatercept to 1.75 mg/kg in up to 35 patients, with 23 responders (TI + HI). The dose-and-epoetin alfa addition-response relation is summarized in **Table 3**. **Table 3** shows the distribution of patients according to the dose applied and the associated response. Included are patients who were treated simultaneously with luspatercept plus epoetin alfa. Epoetin alfa was administered mostly to patients in whom we did not see an appropriate response after reaching the maximum luspatercept dose of 1.75 mg/kg. The maximum dose of 1.75 mg/kg was reached after four cycles. In most of these patients, epoetin therapy was initiated after six to eight applications of luspatercept. There were also 11 patients receiving luspatercept concomitantly with epoetin, which was started prior to luspatercept.

**Table 3:** Relation between dose, the addition of epoetin alfa, and responses.

Dose of luspatercept (mg/kg)	Epoetin alfa added	Number of treated patients	Number of patients who reached TI	The number of patients who reached only HI	Number of nonresponders
1.0	No	11	11		
1.0	Yes	1	1		
1.33	No	3	2		1
1.33	Yes	1	1		
1.75	No	6	3	1	2
1.75	Yes	29	14	5	10

TI, transfusion independence; HI, hematology improvement.

**Figure 2:**
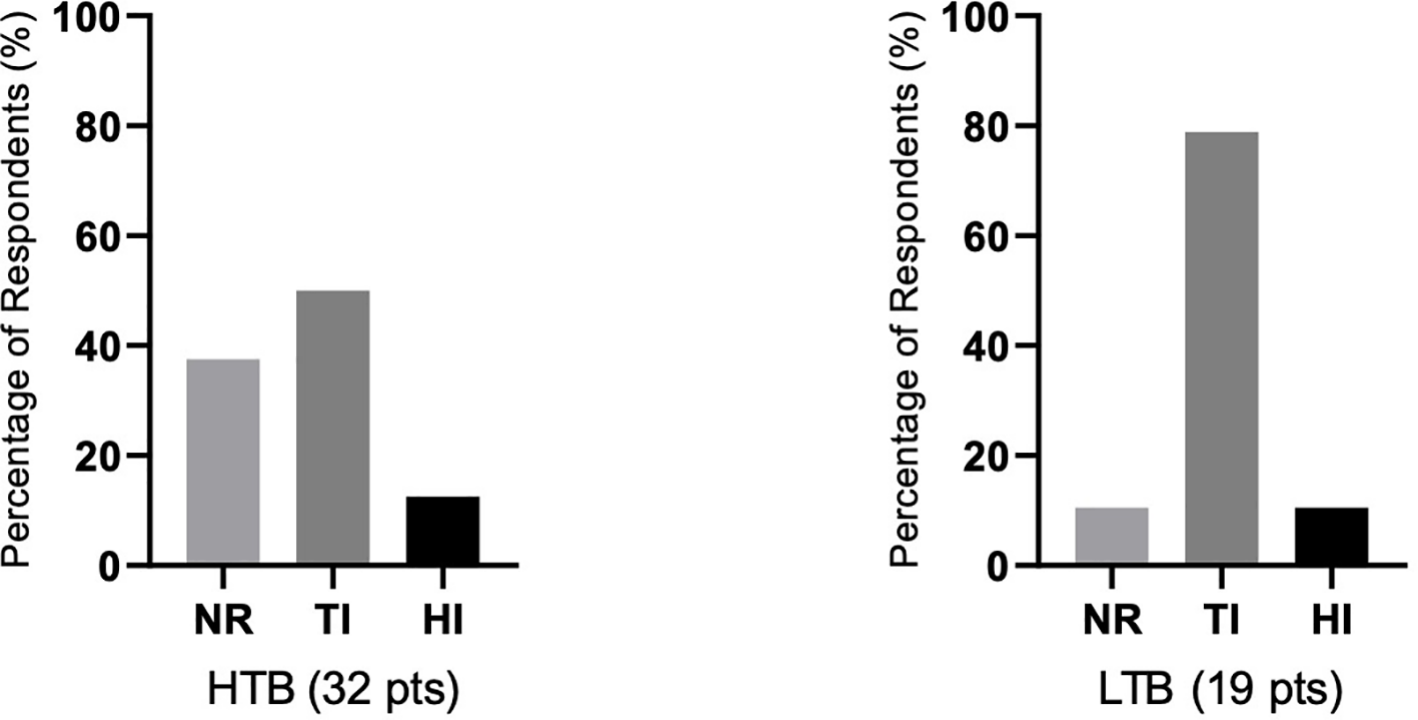
The relationship of responses to therapy in relation to the severity of transfusion dependence prior to therapy. Shown is the percentage of achieving transfusion independence (TI), hematological improvement (HI), or percentage of nonresponders (NR) in both groups of patients with high transfusion dependence/burden ≥ 4 transfusion unit (TU)/8 weeks (HTB, 32 patients) or low transfusion dependence/burden (LTB, 19 patients) with < 4 TU/8 weeks. Assessment refers to the achievement of individual responses lasting ≥ 8 weeks.

**Figure 3:**
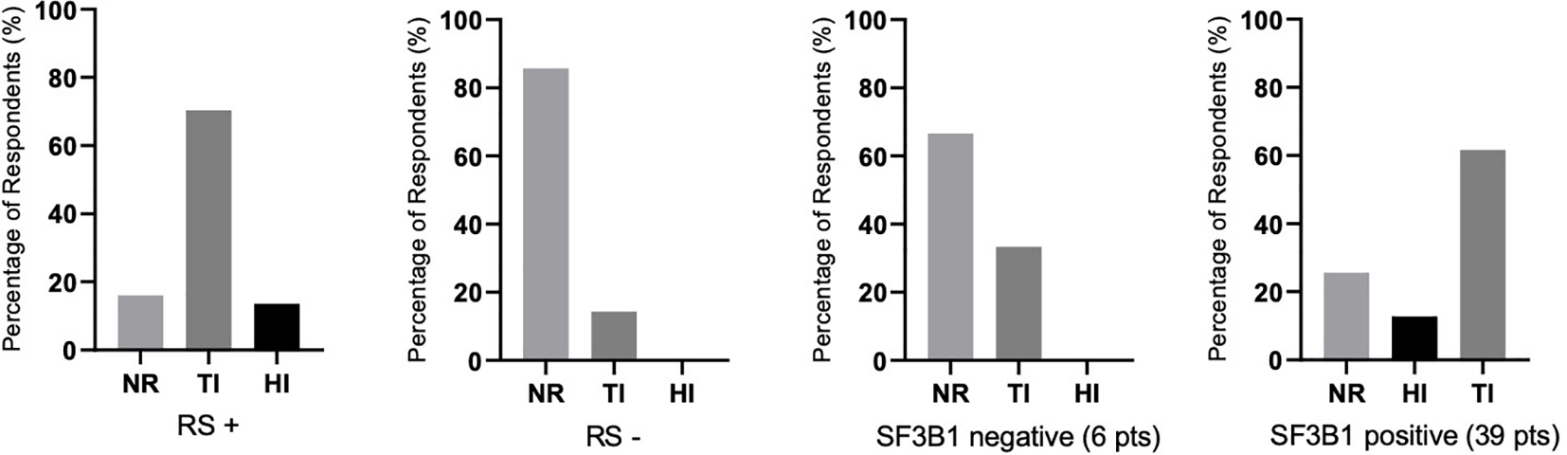
The percentages of responses (assessed by achieving transfusion independence (TI), hematological improvement (HI), or no response (NR) depending on the status of the presence of ring sideroblasts (RS) in the bone marrow (RS+, RS−) and *SF3B1* mutation positivity/negativity (45 patients were evaluated for the presence of the mutation). Assessment refers to the achievement of individual responses lasting ≥ 8 weeks. In RS-negative (RS−) and *SF3B1*-negative groups, there were no patients with HI.

**Figure 4:**
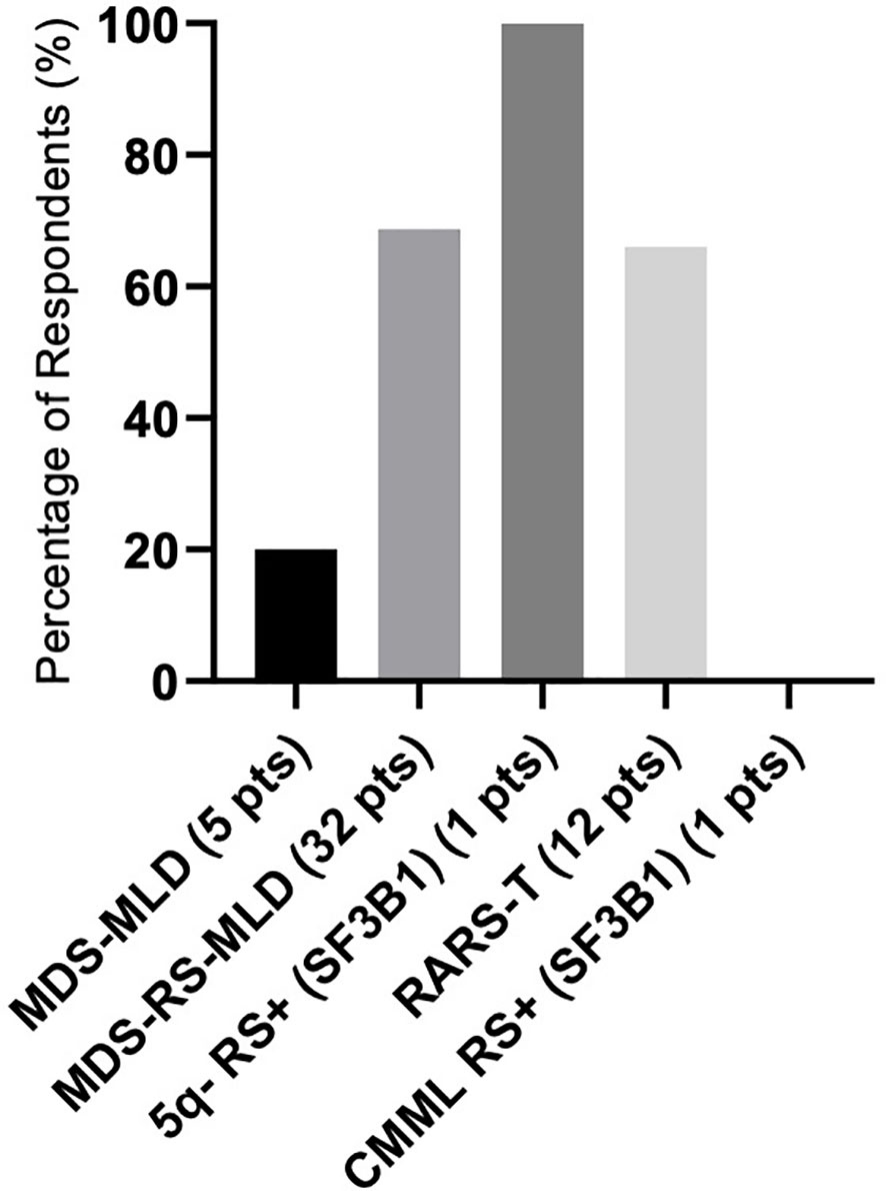
The response rate as represented in the summary of hematological improvement (HI) plus transfusion independence (TI) in each WHO 2016 group. There was one patient with isolated 5q-, ring sideroblasts (RS), and *SF3B1* mutation, and one CMML patient with ring sideroblasts (RS) and *SF3B1* mutation. Assessment refers to the achievement of individual responses lasting ≥ 8 weeks.

**Figure 5:**
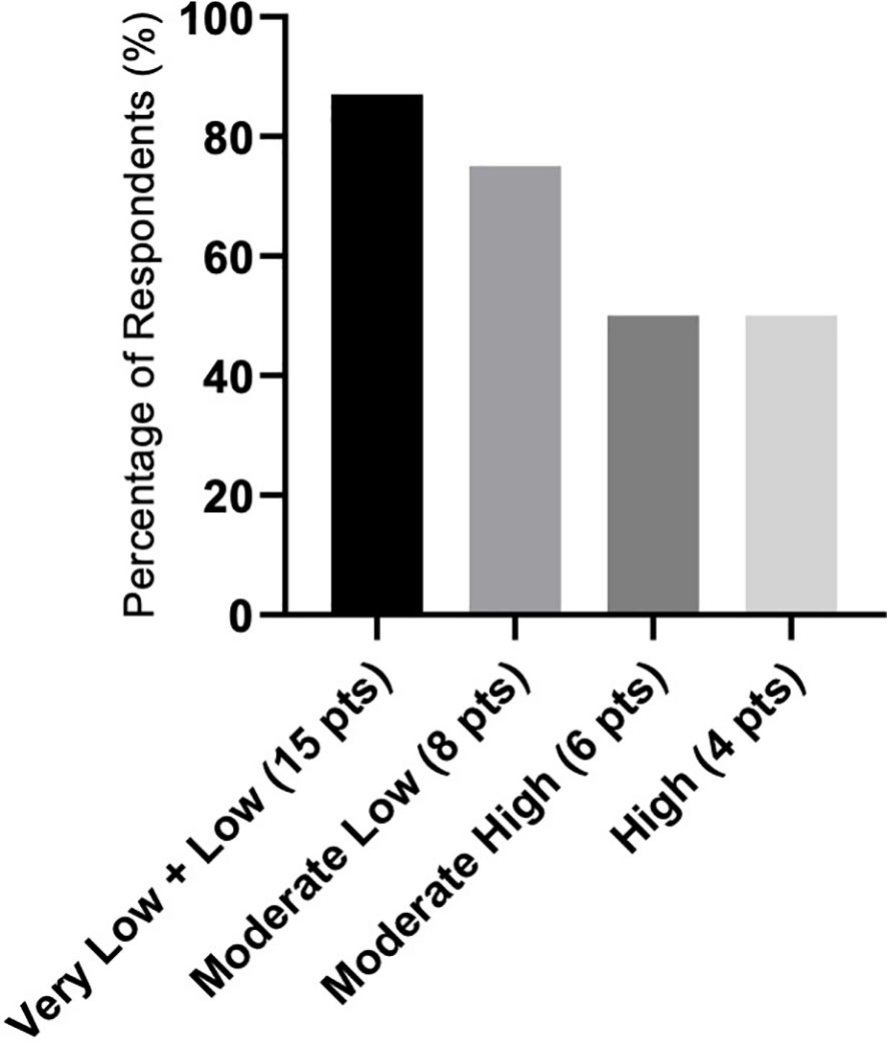
Response rate as represented in the summary of hematological improvement (HI) plus transfusion independence (TI) in each IPSS-M (IPSS molecular) risk group. Assessment refers to the achievement of individual responses lasting ≥ 8 weeks.

There were differences in response according to transfusion burden: in the LTB group (19 patients), 79% reached TI, while in the HTB group (32 patients), 50% reached TI (**Figure 2**). **Figure 3** shows responses in relation to RS status and SF3B1 mutation. In total, 70% of RS+ patients reached TI, while from the small RS− group, only one out of five evaluated patients reached TI. Among 39 *SF3B1*-positive patients, almost 62% responded with TI. **Figure 4** shows the responses (TI+HI) by WHO 2016 classification and **Figure 5** by IPSS-M. For the IPSS-M analysis, for a smaller number of patients with known NGS data, we report the total response, i.e., TI and HI together. There is a relatively significant difference between the IPSS-M categories: in the low and very low IPSS-M group, 87% of patients responded (TI + HI) versus only 50% in higher IPSS-M. This was more significant than in the IPSS-R subgroups. Among the responding patients who achieved TI or HI during treatment, eight (21%) became transfusion-dependent again after a median of 12 months (range, 5–26).

Luspatercept was very well tolerated without any adverse events higher than grade II toxicity. In the course of our analysis, we observed a very limited number of adverse side effects directly related to the initiation of luspatercept therapy, except for transient elevations of blood pressure in three patients, easily resolved by adjusting antihypertensive therapy. No thromboembolic event was present. In the COMMANDS study, reports of fatigue and asthenia were always related only with the degree of anemia experiences by the patients. We did not observe any complications during luspatercept therapy, and the drug was not discontinued in any of the treated patients. Therapy, even in responding patients, was discontinued in 14 patients: five due to progression of MDS, six due to death from other causes, two due to newly diagnosed nonhematological malignancy, and one due to noncompliance.

## Discussion

TGF-β signaling serves as a myelosuppressive factor, impeding erythroid differentiation by inducing apoptosis and halting erythroblast cell cycle progression (7, 13). TGF-β receptor ligands, which include polypeptide growth factors such as TGF-β, activins, bone morphogenetic proteins (BMPs), and GDF11, exert notable effects on hematopoiesis (14). Upon binding to their receptors (activin receptors I and II), these ligands trigger receptor phosphorylation and activate the SMAD pathway. Within the context of TGF-β signaling, SMADs (SMAD2/3 and SMAD1/5/8) play crucial roles in regulating hematopoiesis. In the case of MDS, overactivation of the SMAD2/3 signaling pathways contributes to anemia by impairing terminal erythroid differentiation and maturation (13, 14).

Erythropoietin (EPO) primarily acts on CFU-E progenitors and proerythroblasts to support their survival and facilitate terminal maturation. Additionally, EPO can induce cell proliferation and drive multipotent hematopoietic progenitors toward an erythroid lineage (15, 16). *In vivo* administration of EPO leads to a shift of multipotential progenitors away from the myeloid lineage and toward the erythroid lineage (17). Considering that EPO primarily functions in the early stages of erythropoiesis, promoting the maturation of erythroid progenitors, while luspatercept impacts differentiation in the terminal phase of erythropoiesis by blocking the TGF-β pathway, it is conceivable that these two drugs may exhibit synergistic effects. Furthermore, both drugs are approved for the treatment of anemia in MDS, providing a rationale for combining them in therapy.

This rationale prompted us to add epoetin to luspatercept therapy in patients demonstrating insufficient or slow responses. Our luspatercept-treated cohort exhibits several distinctive features. Firstly, our patients represent an unselected population of lower-risk transfusion-dependent MDS patients encountered in routine clinical practice. Additionally, a small subset of patients with high IPSS-M scores was included in our analysis. Notably, our cohort exhibits a higher prevalence of severe transfusion dependency compared to the populations studied in clinical trials. Secondly, our patients likely have more significant comorbidities, as reflected by the relatively higher number of nonhematologic deaths during the study period.

Our patient population differs from that of the COMMANDS study, primarily due to differences in RS status, with reimbursement limitations allowing treatment only for RS+ patients in our cohort. Moreover, our RS− patients had all undergone previous treatments and exhibited poor responses to multiple lines of therapy, including investigational drugs, predominantly presenting with high transfusion burdens. We believe this contributed to the prevalent resistance to luspatercept observed in this subgroup.

Conversely, we can draw some comparisons between our results in RS+ patients and those of the MEDALIST study. In our RS+ patients, we achieved a higher rate of transfusion independence (70%). We attribute these positive outcomes, albeit in a relatively small patient cohort, to the combination therapy of luspatercept plus epoetin, along with the relatively high percentage of patients receiving the maximum dose of luspatercept.

In cases where a sufficient response was not observed even at the maximum luspatercept dose, the addition of epoetin resulted in a significant increase in response rates, as indicated in **Table 3**. Noteworthy is the case of a patient with 5q- syndrome, previously treated with lenalidomide, who exhibited subsequent therapy failure and was found to have an SF3B1 mutation. Initiation of luspatercept therapy in this patient yielded promising results, with hemoglobin levels reaching 11.0–12.0 g/dL. However, after 12 months, progressive therapy failure was observed once again. Further research is warranted to elucidate the impact of concurrent mutations on treatment responses. Our analysis highlights excellent responders among patients with very low and low IPSS-M scores, though a larger patient cohort is needed to assess the statistically significant influence of other factors, including mutations.

## Conclusion

We have demonstrated in real-world clinical practice that luspatercept is a very effective agent, even in an unselected, pretreated, significantly transfusion-dependent MDS population. The effect was particularly high in the IPSS-M low and very low groups. We believe that the relatively high response rate in our treated population was influenced by the frequent use of a higher dose of luspatercept (1.75 mg/kg) and especially by adding epoetin alfa to luspatercept in insufficiently responding patients.

## Data availability statement

The authors acknowledge that the data presented in this study must be deposited and made publicly available in an acceptable repository, prior to publication. Frontiers cannot accept an article that does not adhere to our open data policies.

## Author contributions

AJ: Conceptualization, Data curation, Formal analysis, Funding acquisition, Investigation, Methodology, Project administration, Supervision, Validation, Visualization, Writing – original draft, Writing – review & editing. SS: Data curation, Writing – original draft. PB: Data curation, Investigation, Writing – review & editing. LM: Data curation, Investigation, Supervision, Writing – review & editing. TS: Investigation, Supervision, Writing – review & editing. JJ: Data curation, Formal analysis, Investigation, Software, Writing – review & editing. TA: Data curation, Investigation, Resources, Writing – review & editing, Formal analysis, Supervision, Validation. ZZ: Data curation, Investigation, Project administration, Resources, Writing – review & editing.
